# Comparison of two different mindfulness interventions among health care students in Finland: a randomised controlled trial

**DOI:** 10.1007/s10459-022-10116-8

**Published:** 2022-05-03

**Authors:** Saara Repo, Marko Elovainio, Eeva Pyörälä, Mónica Iriarte-Lüttjohann, Tiina Tuominen, Tiina Härkönen, Kia Gluschkoff, Tiina Paunio

**Affiliations:** 1 Centre for University Teaching and Learning, University of Helsinki, P.O. Box 21, 00014 Helsinki, Finland; 2 Department of Psychology and Logopedics, University of Helsinki, Helsinki, Finland; 3 MIELI, Mental Health Finland, Helsinki, Finland; 4 Student Services, University of Helsinki, Helsinki, Finland; 5 Research Services, University of Helsinki, Helsinki, Finland; 6 Department of Psychiatry and SleepWell Research Program, University of Helsinki and Helsinki University Hospital, Helsinki, Finland

**Keywords:** Mindfulness, Acceptance and commitment therapy, Health care students, Well-being, Distress, Randomised control trial

## Abstract

We investigated the short- and long-term effects of two different evidence-based mindfulness training on students’ stress and well-being. A randomised controlled trial with three measurement points (baseline, post-intervention, and 4 months post-intervention) was conducted among undergraduate students of medicine, dentistry, psychology, and logopaedics at the University of Helsinki. The participants were randomly assigned into three groups: (1) face-to-face mindfulness training based on the Mindfulness Skills for Students course (n = 40), (2) a web-based Student Compass program using Mindfulness and Acceptance and Commitment therapy (n = 22), and (3) a control group that received mental health support as usual (n = 40). The primary outcome was psychological distress measured using the Clinical Outcomes in Routine Evaluation Outcome Measure (CORE-OM). Secondary outcomes included hair cortisol concentrations and a wide range of well-being indicators. Psychological distress increased in all the groups from baseline to post-intervention, but significantly less so in the intervention groups than in the control group. At 4-month follow-up, were found no differences between the primary outcomes of the control and intervention groups, but the participants who continued practising mindfulness at least twice a week were less stressed than the others. Our results suggest that participating in a mindfulness course may mitigate health care students’ psychological distress during the academic year, but only if the participants continue practising mindfulness at least twice a week.

**Supplementary Information:**

The online version contains supplementary material available at 10.1007/s10459-022-10116-8.

## Introduction

High levels of psychological stress have been reported among students of health care professions, such as students of medicine, dentistry and psychology (Dahlin et al., 2005; Dyrpye et al., 2005; Jain, 2012). The concept of psychological stress is ambiguous and may refer to multiple stages of the whole stress process, including environmental threats, appraisal of particular stressors, various environmental stress factors, physiological stress responses and related psychological states such as fear, anxiety and distress. The concept has developed since the early ‘fight-or-flight response’ studies (Cannon, 1915), and the current view argues that psychological and physiological stress responses arise when people perceive that demands from the environment exceed their resources or adaptive capacity (McEwen, 2000; McEwen et al., 2015). Demands or stressors include factors such as acute stressful life events (sickness in the family or death of a loved one) and long-term challenges (chronic work-related stress or study pressures and daily minor harassments). Psychosocial resources include social support, the cognitive and physiological ability to cope with challenges, and various personality characteristics. Stress produces a range of physiological changes through multiple biological systems including the hypothalamic–pituitary–adrenocortical and sympatho–adrenomedullary axes and activates the adrenal cortex to initiate the synthesis and release of glucocorticoid hormones such as cortisol, promoting the mobilisation of stored energy (Ulrich-Lai & Herman, 2009).

Moderate and short-term stress may not negatively affect health, but extreme levels of psychological stress and chronic stress have shown to have negative effects on performance and health (McEwen et al., 2015). Effective stress management skills may ease students’ academic performance and professional lives and may enhance their well-being. In recent decades, various mindfulness-based interventions have been developed and studied in health profession education (de Vibe et al., 2013, 2018; Gilmartin et al., 2017; Ludwig & Kabat-Zinn, 2008; O’Driscoll et al., 2017; Orosa-Duarte et al., 2021; Pinnock et al., 2021; Rosenzweig et al., 2003; Warnecke et al., 2011;).

Mindfulness can be defined as a specific skill of intentionally focusing on the present moment and observing it with acceptance. The object of non-judgemental attention can be either internal, such as bodily sensations, emotions, and thoughts, or external, such as other human beings or the physical environment, or the interaction between the internal and external world, for example, the reactions that other people arouse in us (Burton et al., 2017; Farb et al., 2007; Kabat-Zinn et al., 1992). This present-centred self-awareness skill can be developed through regular practice and it promotes ways in which to respond to internal reactions by improving awareness, attention regulation, and acceptance without the need to invest in, change or escape from them (Cho et al., 2016; Farb et al., 2007).

Jon Kabat-Zinn and his colleagues (Kabat-Zinn, 2013; Santorelli, 2014) developed an eight-week Mindfulness-based Stress Reduction (MBSR) programme, which is the most researched mindfulness programme for both the general population and health care students (McConville et al., 2017 and Burton et al., 2017). Mindfulness-based Cognitive Therapy (MBCT), developed in the UK especially for depressed patients, has also been applied to interventions among higher education students (Galante et al., 2018). According to two systematic reviews (McConville et al., 2017 and Burton et al., 2017), mindfulness-based interventions prevent experiences of stress, decrease anxiety (McConville et al., 2017), protect from depression (McConville et al., 2017; Mascaro et al., 2018; Shapiro et al., 1998), increase resilience (Galante et al., 2018) and increase self-regulation (de la Fuente et al., 2018) among health care students. In addition, mindfulness training may improve social and emotional skills such as empathy (McConville et al., 2017) for future health care professionals.

Acceptance and Commitment Therapy (ACT), a recently developed intervention designed to improve psychological flexibility (Hayes et al., 2011) supports participants to find their values, commit to value-based actions, follow their thinking (eg. Engage Components) and be present and aware, opening to acceptance and contact with present moment (eg. Mindfulness Components). There is evidence that a seven-week ACT intervention can reduce stress among university students in different fields (Räsänen et al., 2016). Even a four-week intervention has been found to be effective (Grégoire et al., 2018; Viskovich & Pakenham, 2020). However, the results are inconsistent with respect to the role of the engage component and the mindfulness component in stress management. (Levin et al., 2020; Morin et al., 2021). Although there is evidence that ACT can reduce stress among postgraduate students in clinical psychology (Pakenham & Stafford-Brown, 2013) and university students in various fields (Räsänen et al., 2016), more research is needed on its effectiveness among academic health care students.

The implementation of mindfulness-based and ACT programmes varies considerably, and it may be challenging to determine the key cornerstones of an effective intervention (Burton et al., 2017). It seems that the most important aspect is that participants practise mindfulness on a regular basis (Correia et al., 2017). To do this, they need easy access to guidelines for mindfulness meditation and support for regular personal practice. Participants benefit from strong support, especially at the beginning of the process, when they are likely to face their distress and reactivity (Monshat et al., 2013).

Previous research suggests that a mindfulness teacher can provide effective support either in groups or individually. Peer group learning, mobile applications, traditional workbooks, and individual phone calls can also facilitate learning (Burton et al., 2017; McConville et al., 2017). According to Hazlett-Stevens and Oren (2017), Mak et al. (2017) and Orosa-Duarte et al. (2021), web-based mindfulness interventions have been effective. According to Viskovic and Pakenham (2020), Kraft et al. (2019), and Levin et al. (2021) similar findings have been made for ACT interventions, but the dropout rates have been high. Individual workbook learning (Hazlett-Stevens & Oren, 2017) has the same disadvantage, but regular support over the phone (Mak et al., 2017) or from a peer student ACT coach (Räsänen et al., 2016) may keep up motivation. McConville and her colleagues (2017) emphasise the importance of introducing a variety of mindfulness practice options (body scanning, mindful meditation, and mindful movement), so that participants find the most appropriate practice methods. More research is needed on the effectiveness of different mindfulness training methods including ACT interventions as well as their long-term impact on students’ stress management (O’Driscoll et al., 2017). Further randomised controlled trials are also needed to determine the effects of mindfulness interventions (de Vibe et al., 2013; Gilmartin et al., 2017; Pinnock et al., 2021) and ACT interventions (Levin et al., 2020; Morin et al., 2021) With the Covid-19 pandemic, we have learned that we need to develop both face-to-face and online versions of mindfulness training.

We investigated the short- and long-term effects of face-to-face mindfulness and web-based ACT training on the stress and well-being of students of a variety of academic health service fields, including medicine, psychology, logopaedics and dentistry. As outcome variables, we used several psychological survey measures, and we also measured the participants’ long-term stress hormone (cortisol) levels from hair samples (Stadler & Kirschbaum, 2012). To date, only a few studies have assessed the effect of mindfulness interventions and none of ACT interventions by measuring participants’ cortisol levels (Lamothe et al., 2020).

The aim of this study was to test whether two types of mindfulness interventions, face-to-face mindfulness and web-based ACT training, could enhance students’ well-being. Our specific research questions were: (1) What were the short- and long-term effects of mindfulness interventions on students’ stress and well-being? (2) Were the effects of the face-to-face mindfulness and web-based ACT interventions different compared to the control group? (3) How did the interventions motivate participants to practise mindfulness independently during the course?

## Materials and methods

### Recruitment

**Fig. 1:**
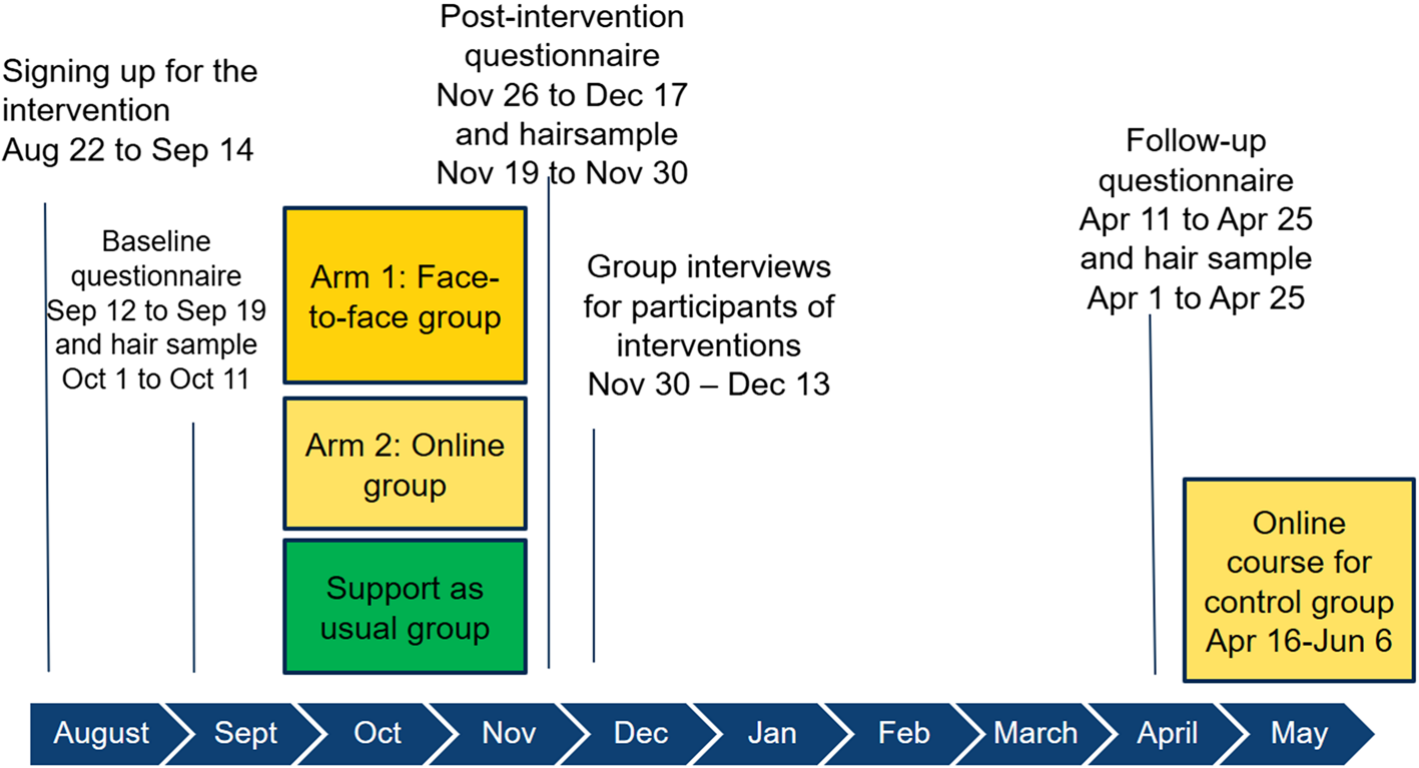
Trial timeline August 2018–June 2019

**Fig. 2:**
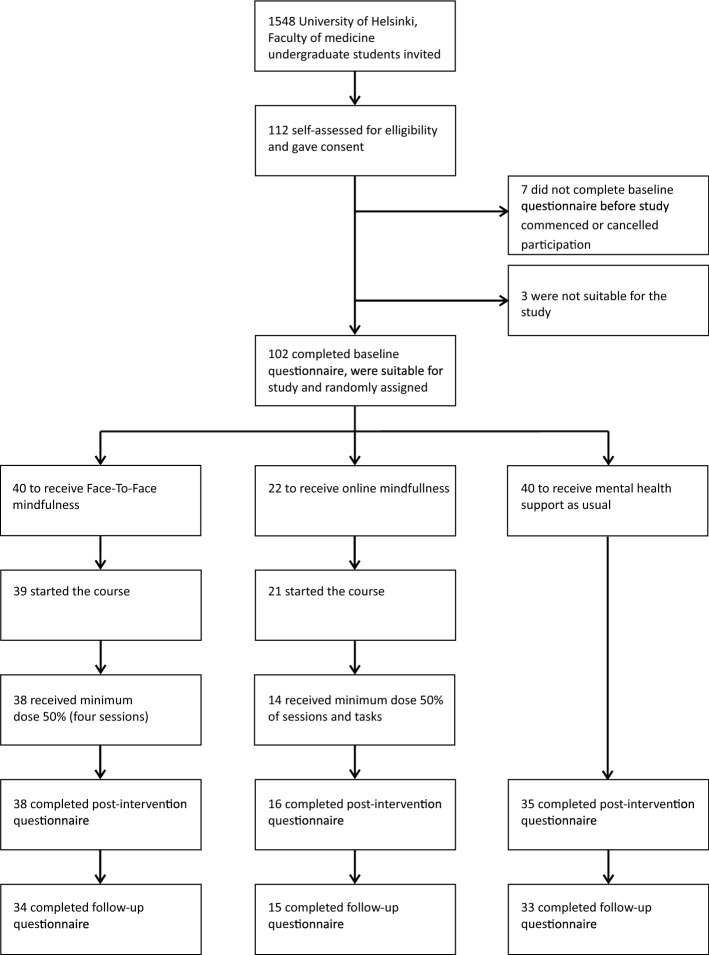
Trial profile

A randomised controlled trial was conducted among undergraduate students of medicine, dentistry, psychology and logopaedics at the University of Helsinki. We emailed all undergraduate students who had begun their studies in 2009 or later at the Medical Faculty to invite them to participate (N = 1548). The students were informed that participation was voluntary and that they were allowed to withdraw from the study at any point of the trial. Figures 1 and 2 provide more details on the procedure and trial profile. We included all those who enrolled and agreed, by signing the consent format, to make a full contribution to the intervention (to participate in group meetings and commit to doing the daily training) irrespective of the group into which the participant was randomly placed. The exclusion criteria were defined as follows: participants who had severe mental problems (such as anxiety or depression) at the start of the study, who had suffered a major loss or trauma in their recent past, who had any other mental or physical health problem that could make participation difficult, or who were receiving ongoing psychotherapy. Three participants were excluded based on their responses to the baseline questionnaire. They were offered the same online ACT course as the control group, i.e., the normal support offered to students. The eligibility criteria were assessed by the students themselves and by a committee that consisted of a medical doctor from the student health care centre and two members of the research group.

### Study protocol registration, approval, and funding

The study protocol was registered in ClinicalTrials.gov before the study began and accepted on September 12, 2018 (identifier number is NCT03669016). The Helsinki University Hospital Ethics Committee approved the trial on August 10, 2018. The study was funded by the Faculty of Medicine of the University of Helsinki. The members of the research group conducted the research as part of their academic work. The study plan was approved by the Faculty of Medicine management team.

### Data collection

Survey data on the demographics and well-being outcomes were collected three times using an electronic questionnaire: at baseline, post-intervention and after a 4-month follow-up. The start of the interventions and baseline data collection was scheduled for the beginning of the academic year in September (Fig. 1). The interventions ended at the end of November and collecting of the post-intervention data started a week later. We wanted, firstly, to study the immediate effects of the interventions, and secondly, ensure that participants answered the questionnaire before they left for their well-earned vacation after the end of busy first semester. We decided to collect the follow-up data at the end of the second semester, because that is typically another stress point for the students. A personal link to the survey was emailed to the participants.

Hair samples were collected on separate occasions. The procedure followed the instructions of the Faculty of Science, Department of Psychology, Technische Universität Dresden. Two hair strands (approximately 5 mm thick) were taken from as close to the scalp as possible. The samples were wrapped in aluminium foil, marked with a personal identification number, and stored in dark, dry place. They were collected at baseline, post-intervention and after the 4-month follow-up. All the hair samples were sent to the Laboratory of Biopsychology, Technishe Universität Dresden, immediately after the last measurement, at the end of April 2019. We analysed the samples between May 8 and Jun 6.

### Randomisation

The participants who filled in the baseline questionnaire and were eligible for the study were randomised using the Sampling package (Tillé & Matei, 2016) of R software version 3.5.2 (R Core Team, 2018). The participants were randomly assigned into three groups to receive either (1) face-to-face mindfulness training using the Mindfulness Skills for Students course plus mental health support as usual (n = 40), (2) a web-based Acceptance and Commitment therapy training using the Student Compass course plus mental health support as usual (n = 22), and (3) a control group with mental health support as usual (n = 40). We initially aimed to recruit 150 participants but enrolled only 102 eligible participants. Based on the effect size calculation reported in study protocol, the sufficient group size would have been 35 participants. Because it could be presumed that some of participants would discontinue before the end of the intervention, we wanted to make sure that at least one of the intervention arms would have enough participants for valid statistical analyses.

Randomisation was stratified based on demographic variables (gender, age and study programme). Each participant was informed by email of their allocation in one of the two intervention groups or a control group and was asked to confirm their participation in the study by email.

### Procedures

#### Control group: student support services as usual

The control group received the usual support offered to students on campus. Two of the University of Helsinki’s study psychologists work on the medical campus and provide students with support for study difficulties and refer those who need more support to the student health care services. Control group participants were offered an online ACT course after the follow-up measurement.

#### Interventions

We tested two different mindfulness interventions to find feasible ways to lower specifically academic health care students’ distress and give them possibility to cultivate capacity to handle stress in their future work as well. The following programs were chosen, because they had proven to be effective among university students: (1) Mindfulness Skills for Students program was turn out to lower distress of higher education students in Cambridge University, United Kingdom context (Galante et al., 2018), (2) the Student Compass created in the University of Jyväskylä, Finland, was a web-based wellness and life skills program, developed originally in Finnish language and available at the beginning of the study. The Student Compass online course had been successfully implemented in a previous study of a university student population (Räsänen et al., 2016), and was therefore seen as a valid online method for teaching mindfulness skills to students of the same age.

The intervention courses were free for the participants. If a participant answered the questionnaires and provided hair samples at all three measurement points, they received two cinema tickets. No other compensation (such as study credits) was provided. The interventions had different facilitators, both were licensed clinical psychologists and specialised in student counselling. In addition, the facilitator of the face-to-face mindfulness group was trained in mindfulness and the facilitator of the online ACT group was trained in the theory and methodology of Acceptance and Commitment Therapy.

The two interventions in our study differed in three ways: (1) the face-to-face mindfulness course had a mindfulness teacher who met the students once a week for eight weeks, whereas the online ACT course participants met the teacher face-to-face only twice, at the beginning and at the end of the intervention. (2) In the face-to-face mindfulness course, the students formed two coherent groups that worked together. On the online ACT course, some participants reported forming spontaneous pairs because they knew each other in advance, but participation in the programme was mainly solitary. (3) The face-to-face mindfulness course participants followed the MBTC-based Cambridge Mindfulness for Students programme, which concentrated on various aspects of mindfulness. The online course participants followed the ACT-based programme, where aim was to learn mindfulness as well as other skills of psychological flexibility. It could be presumed that the MBCT programme would increase mindfulness skills to a greater extent than the ACT-based programme, in which mindfulness exercises were only part of the programme.

A detailed description of the two different intervention arms and a comparison between them is presented in the Table 1.

**Table 1:** Description and comparison of two different intervention arms

	Arm 1: Face-to-face group-based mindfulness training	Arm 2: Web-based mindfulness training
Theoretical background	Mindfulness Based Cognitive Therapy and Mindfulness Based Stress Reduction courses	Acceptance and Commitment therapy
Main publication	Galante, J., Dufour, G., Vainre, M., Wagner, A. P., Stochl, J., Benton, A., Lathia, N., Howarth, E., & Jones, P. B. (2018). A mindfulness-based intervention to increase resilience to stress in university students (the Mindful Student Study): a pragmatic randomised controlled trial. The Lancet. Public health, 3(2), e72–e81 (Galante et al. 2018)	Räsänen, P., Lappalainen, P., Muotka, J., Tolvanen, A., & Lappalainen, R. (2016). An online guided ACT intervention for enhancing the psychological wellbeing of university students: A randomized controlled clinical trial. Behaviour Research and Therapy, 78 (March), 30–42. (Räsänen et al. 2016)
The aim of the intervention	Reduce harmful distress	Increase psychological well-being and flexibility
Number of face-to-face meetings	Eight meetings that lasted 75–90 min	Opening and closing meeting that lasted 60 min
Durance of the program	Eight weeks	Eight weeks
Replication of	Mindfulness Skills for Students program created in the University of Cambridge, UK	Student Compass program created in the University of Jyväskylä, Finland
Content of the intervention	Noticing automatic patterns and cultivating mindful awareness, connecting with the body; maintaining attention in the present with acceptance and compassion; motivation and intention; bringing awareness to internal speech; moving towards difficulties rather than away from them; awareness of thoughts, emotions, impulses to act and bodily sensations and relating to them as passing events in the mind and body; including and doing enjoyable things in life, living life whole heartedly; and maintaining and expanding the practice of mindfulness	The concept and research of psychological flexibility, finding your values, committing to value-based actions, being present and aware, following one’s thinking, opening to acceptance and contact with the present moment
Stucture of the program and type of sessions	The sessions followed the same structure throughout the course to ensure familiarity and safety. They started and ended with meditation, and participants shared their experiences in pairs, small groups or with the whole group. Each session had a main theme and different meditation practices, followed by weekly home readings and formal and informal meditation practices	The opening session: Introduction to the concept and research on psychological flexibility and detailed instructions for the Student Compass program and online environment The students were encouraged to practice mindfulness and psychological flexibility skills at home and to report their findings weekly. They shared their self-study findings and answered short essay questions on an online platform designed to clarify learning and to monitor progress. The closing session: the participants could ask questions and reflect on their progress They received additional instructions from the teacher, shared their experiences with others, and provided feedback
Physical environment of the sessions	A normal classroom on campus, which was furnished with carpets, cushions, and blankets	A normal classroom on campus
Type of materials	A book (translated into Finnish) Williams & Penman (2011) and audio recordings connected to the book were available in internet and in Finnish language. www.viisaselama.fi/mp3/tietoinenlasnaolo The teacher also used a manual written at the Cambridge University: Mindfulness Skills for Students (English, 2016)	Largely based on texts, but also several audio recordings of excercises, self-study questions, examples of student life and videos in Finnish language. Available in Student Compass web environment
Practices participants were supposed to do on their own	Various mindfulness practices (body scan, breathing, sounds, movement, loving kindness) 10–30 min per a day	Various mindfulness and reflective practices, a weekly short reflective essay of their findings
Teacher’s role	Introduce the theme of the week, guide the meditations, encourage students to explore their experiences in class and during the week in an accepting way, as well as share their experiences in class in small groups and with the whole group. Be available for participants personal questions after the weekly sessions	Lead the opening and closing sessions, communicate with the students on the online platform, and give personal written feedback in the middle of the course to each participant and to the group

### Outcomes

The primary outcome measure was psychological distress, examined using Clinical Outcomes in Routine Evaluation Outcome Measure (CORE-OM). The CORE-OM includes four domains: subjective well-being, symptoms, functioning, and risks. The items in each domain are rated on a scale from 1 to 5, with higher scores indicating higher distress. A mean score was calculated for each domain. Number of items, scales and Chronbach’s Alphas are presented in Table 2. The measure was chosen because it is suitable for indicating patients’ responses to psychotherapeutic treatment, a validated tool for examining the effects of psychosocial interventions (Evans et al., 2002) and was used in Cambridge University Mindfulness intervention study as well (Galante et al., 2018).

**Table 2:** Number of items and Chronbach’s Alphas of primary outcome of CORE-OM and its four domains: well-being, symptoms, functioning, risk

	No of items	Alpha
CORE-OM total	34	0.8874
CORE-OM well-being	4	0.6524
CORE-OM symptoms	12	0.7945
CORE-OM functioning	12	0.7087
CORE-OM risk	6	0.6837

There were two types of secondary measures: biological and psychological. The biological indicator of stress was cortisol measured from hair samples (Stadler & Kirschbaum, 2012). The questionnaire also included numerous secondary psychological measures of stress and well-being which were used in faculty’s well-being survey conducted 2018. They provide more detailed information of the possible effects of the interventions but were not in the main focus of this article, and therefore they are described in the supplement.

In addition, in the post-intervention survey, we asked the participants ‘How often did you practise mindfulness or meditation in the last 2 months?’ and ‘How long did you practise mindfulness each time?’.

### Power calculations and analyses

Before the study started, we conducted power calculations for determining group sizes based on the study by Connell et al. (2007), which reported the reliable change index of CORE-OM measure as being 3.6 (mean in normal population 4.8, and in low psychological distress population 2.5, with corresponding standard deviations of 4.3 and 1.4). These figures produced effect sizes (Cohen’s d) of 0.79 and 0.72. These effect sizes, with a p < 0.05 statistical significance level and 90% power level, indicated that 35 to 43 would be the optimal group size. The data were analysed using repeated measures mixed modelling and the Stata 15 program. Repeated measures mixed model was chosen because the data require an appropriate accounting for correlations between the observations made on the same subject and possible heterogeneous variances among observations on the same subject over time. Repeated measurements from a particular subject are likely to be more similar to each other than measurements from different subjects and this correlation needs to be considered in the analysis of the resulting data. Many common statistical methods, such as linear regression models, should not be used in this situation because those methods assume measurements to be independent of one another.

## Results

### Participants and their representativeness of the population

We recruited 102 students (74 female, 26 male and 2 undisclosed) from all the five study programmes of the faculty. At baseline, 29.4% of the participants experienced ‘quite a lot’ or ‘a lot’ of stress.

The participants closely represented the population of undergraduate students in the faculty in terms of gender, age, and study year. Students of psychology and logopaedics were over-represented in the data, whereas those of medicine and dentistry were under-represented (Table 3). In addition, compared to the students from the Faculty of Medicine who had participated in a well-being survey in the spring of 2018 (N = 845), the participants of this study experienced less stress on average (M = 3.06, SD = 0.89 vs. M = 3.45, SD = 1.06, t(945) = -4.400, p < 0.001) and had a higher level of self-perceived functional ability (M = 7.77, SD = 1.27 vs. M = 7.06, SD = 2.34, t(943) = 4.815, p < 0.001) (Supplement Table S1).

**Table 3:** Characteristics of participants after randomization

	Support as usual	Face-to-face	Online	All	Proportion of population %	Population /group invited N
N	40	40	22	102	6.6%	1555
*Sex*						
Female	29 (72.5%)	28 (70%)	17 (77%)	74 (72.5%)	7.4%	1006
Male	11 (27.5%)	11 (27.5%)	4 (18.2%)	26 (25.5%)	4.7%	549
Other		1 (2.5%)	1 (4.5%)	2 (2%)	–	–
*Age*						
19–24	20 (50%)	19 (46.2%)	11 (50%)	50 (48.5%)	5.7%	874
25–30	13 (32.5%)	13 (33.3%)	7 (31.8%)	33 (32.7%)	3.9%	485
> 30	7 (17.5%)	8 (20.5%)	4 (18.2%)	19 (18.8%)	9.7%	196
*Beginning of studies (study year)*						
2018 (1st year)	7 (17.5%)	9 (22.5%)	4 (18.2%)	20 (19.6%)	6.8%	292
2017 (2nd year)	10 (25%)	11 (27.5%)	10 (45.5%)	31 (30.4%)	11.3%	274
2016 (3rd year)	4 (10%)	8 (20%)	2 (9.1%)	14 (13.7%)	5.7%	246
2015 (4th year)	11 (27.5%)	5 (12.5%)	4 (18.2%)	20 (19.6%)	4.9%**	743 (2009–2015)
2014 (5th or more)	8 (20%)	7 (17.5%)	2 (9.1%)	17 (16.7%)	**	*
*Program*						
Dentistry	3 (7.5%)	1 (2.5%)	1 (4.5%)	5 (4.9%)	1.8%	273 (17.6%)
Logopaedics	4 (10%)	4 (10%)	7 (31.5%)	15 (14.7%)	11.5%	130 (8.4%)
Medicine	12 (30%)	14 (35%)	9 (40.9%)	35 (34.3%)	4.4%	789 (51%)
Psychology	20 (50%)	21 (52.5%)	5 (22.7%)	46 (45.1%)	13.9%	330 (21.3%)
Translational medicine	1 (2.5%)	–	–	1 (1%)	3.8%	26 (1.7%)
*Previous meditation experience*						
No experience	18 (45%)	20 (50%)	14 (63.6%)	52 (51%)		–*
Experience	22 (55%)	20 (50%)	8 (36.4%)	50 (49%)		–*
Less than 6 months	15 (37.5%)	13 (32.5%)	4 (18.2%)	32 (31.4%)		–*
Six months or more	7 (17.5%)	7 (17.5%)	4 (18.2%)	18 (18.6%)		–*

*Data missing

### Participation in the interventions

The participation rate was excellent in the face-to-face mindfulness group (average participation rate 88%). A total of 38 participants (95%) were exposed to the minimum amount of mindfulness (i.e., more than 50% of the sessions, defined in the Clinical Trial Protocol). The participation rate of the online ACT course was also very good (66%). A total of 63% (14) of the students participated in at least 50% of the programme. In the control group, five participants dropped out before the post-intervention questionnaire and two more before the follow-up questionnaire. Thus, altogether 83% of the control group members took part in the entire study. Both interventions motivated participants to practice mindfulness on their own during the course. Practice frequency and length were highest in the face-to-face mindfulness group, second highest in the online ACT group. In addition, almost one third of the control group did practice mindfulness during the intervention. Tables 4 and 5 provide more details.

**Table 4:** Frequency of individual mindfulness practice during intervention

	None	Less than once a week	Once a week	2–3 Times a week	Almost daily	Total
Support as usual	24	4	4	2	1	35
Face-to-face	0	0	6	17	15	38
Online	0	3	3	8	2	16
Total	24	7	13	27	18	89

**Table 5:** Length of single individual mindfulness practice session during intervention

	Not at all	< 10 min	10–30 min	> 30 min	Total
Support as usual	24	5	6	0	35
Face-to-face	0	12	26	0	38
Online	0	16	0	0	16
Total	24	33	32	0	89

### Comparison of two different interventions to the control group

The control group (n = 38) was compared to the face-to-face mindfulness (n = 39) and online ACT (n = 20) groups separately, and to both intervention groups pooled together (n = 59). The analyses detected the following trend: the difference between the control group and the face-to-face mindfulness intervention group was frequently statistically more significant than the difference between the control and the online ACT group. When the intervention groups were pooled together, the statistically significant difference was about the same as that for the face-to-face mindfulness group alone. Next, we report the exact results.

### Immediate effects on psychological distress and other measures

Psychological distress (primary outcome measure CORE-OM) increased in all the groups from baseline to post-intervention, but this increase was smaller in the intervention groups than in the control group (Tables 6 and 7). The differences between the control group and intervention groups were not, however, statistically significant (p = 0.076 for the difference between control group and face-to-face mindfulness group; p = 0.516 for the difference between control group and online ACT intervention; p = 0.067 for the difference between control group and pooled intervention groups). However, in the CORE-OM domain of ‘functioning and social interaction’, the effect of the interventions was more pronounced. The difference was significant between the control group and face-to-face mindfulness group alone (p = 0.028), and between the control groups and both intervention groups pooled together (p = 0.028).

**Table 6:** Baseline, post-intervention and follow-up measurements of primary outcome of CORE-OM and its four domains: well-being, symptoms, functioning, risk; and secondary outcome of hair samples

	Baseline			Post-intervention			Follow-up		
	n	Mean (SD)	Median (min– max)	n	Mean (SD)	Median (min–max)	n	Mean (SD)	Median (min–max)
*CORE–OM total*									
Support as usual	38	1.67 (.27)	1. 68 (1.24–2.24)	38	1.78 (.35)	1.75 (1.18–2.53)	34	1.82 (.35)	1.79 (1.29–2.74)
Face-to–face	39	1.70 (.32)	1.65 (1.24– 2.65)	35	1.94 (.37)	1.91 (1.29–2.68)	33	1.81 (.39)	1.82 (1.29– 2.68)
Online	20	1.63 (.33)	1.57 (1.18– 2.44)	16	1.79 (.35)	1.79 (1.24–2.41)	15	1.82 (.33)	1.91 (1.29– 2.29)
*CORE-OM well-being*									
Support as usual	38	1.78 (.40)	1.75 (1–3)	38	1.91 (.56)	1.75 (1.00–3.00)	34	1.93 (.56)	1.75 (1.00–3.50)
Face-to-face	39	1.90 (.58)	1.75 (1–3.5)	35	2.20 (.56)	2.25 (1.25–3.25)	33	2.05 (.66)	2.00 (1.00–3.75)
Online	20	1.7 (.50)	2.00 (1–3)	16	1.86 (.52)	1.88 (1.00–3.00)	15	1.90 (.51)	2.00 (1.25–2.75)
*CORE-OM symptoms*									
Support as usual	38	1.77 (.39)	1.79 (1–2.42)	38	1.94 (.53)	1.79 (1.08–3.00)	34	2.00 (.53)	1.83 (1.17–3.25)
Face-to-face	39	1.82 (.44)	1.75 (1.25–2.83)	35	2.15 (.52)	2.08 (1.17–3.00)	33	1.97 (.52)	1.92 (1.17–3.00)
Online	20	1.74 (.46)	1.67 (1.08–2.83)	16	1.99 (.5)	1.92 (1.17–3.00)	15	2.00 (.4)	2.08 (1.33–2.58)
*CORE-OM functioning*									
Support as usual	38	1.80 (.34)	1.75 (1.33–2.50)	38	1.85 (.35)	1.83 (1.25–2.92)	34	1.88 (.34)	1.92 (1.33–2.75)
Face-to-face	39	1.76 (.32)	1.75 (1.33–2.67)	35	1.99 (.44)	2.00 (1.33–3.17)	33	1.86 (.41)	1.75 (1.33–2.75)
Online	20	1.74 (.36)	1.67 (1.25–2.5)	16	1.86 (.43)	1.83 (1.08–2.50)	15	1.93 (.46)	1.92 (1.08–2.75)
*CORE-OM risk*									
Support as usual	38	1.01 (.04)	1 (1–1.17)	38	1.01 (.04)	1.00 (1.00–1.17)	34	1.01 (.04)	1 (1.00–1.17)
Face-to-face	39	1.03 (.08)	1 (1–1.33)	35	1.02 (.05)	1.00 (1.00–1.17)	33	1.02 (.05)	1 (1.00–1.17)
Online	20	1.01 (.04)	1 (1–1.17)	16	1.03 (.13)	1.00 (1.00–1.5)	15	1.01 (.04)	1 (1.00–1.17)
*Hair samples*									
Support as usual	34	10.46 (6.70)	9.52 (1.48–26.81)	30	9.62 (15.84)	5.30 (1.24–87.62)	30	5.74 (2.99)	5.43 (0–12.64)
Face-to-face	37	15.26 (15.49)	10.1 (2.81–76.00)	35	9.86 (17.59)	5.40 (0–103.47)	32	8.46 (12.54)	4.76 (1.17–55.13)
Online	19	11.53 (11.04)	7.5 (3.12–49.72)	15	5.53 (3.96)	4.31 (0–13.06)	13	4.27 (2.91)	3.77 (0–11.51)

**Table 7:** P-values of repeated measures mixed model, immediate and longitudinal effects of primary outcome CORE-OM and its four domains: well-being, symptoms, functioning, risk, and secondary outcome of hair samples

	Immediate effect	Longitudinal effect
*CORE–OM total*		
Support as usual vs. face-to-face	0.076	0.516
Support as usual vs. online	0.242	0.484
Support as usual vs. both interventions	0.067	0.424
*CORE-OM well-being*		
Support as usual vs. face-to-face	0.162	0.841
Support as usual vs. online	0.294	0.828
Support as usual vs. both interventions	0.137	0.796
*CORE-OM symptoms*		
Support as usual vs. face-to-face	0.159	0.411
Support as usual vs. online	0.348	0.541
Support as usual vs. both interventions	0.144	0.374
*CORE-OM functioning*		
Support as usual vs. face-to-face	0.028	0.997
Support as usual vs. online	0.191	0.430
Support as usual vs. both interventions	0.028	0.727
*CORE-OM risk*		
Support as usual vs. face-to-face	0.471	0.372
Support as usual vs. online	0.130	0.494
Support as usual vs. both interventions	0.244	0.332
*Hair samples*		
Support as usual vs. face-to-face	0.194	0.601
Support as usual vs. online	0.219	0.539
Support as usual vs. both interventions	0.145	0.540

We found no evidence that the interventions influenced cortisol levels measured from hair samples. The average level of cortisol decreased in all the participants from baseline to follow-up and the change was statistically significant (p = 0.049), but there were no differences between the groups (Tables 6 and 7).

Some immediate effects of face-to-face mindfulness group on secondary outcomes were found: stress (measured using one question) (p = 0.02), functional ability (p = 0.037), and study load (0.056). We did not find the same trends in the online ACT group. We also found no evidence that the interventions had any effect on quality of life, psychological flexibility, recovery, resilience, self-perceived health, mental well-being. There were some effects on personality traits. Neuroticism increased from baseline to post-intervention in the control group but did not change in the intervention groups. The difference was significant (p = 0.013) with the face-to-face mindfulness group. No immediate differences in conscientiousness were detected. Supplement Table S2 presents the key figures of the secondary outcomes and Supplement Table S3 presents all p-values of secondary outcomes.

### Longitudinal effects on psychological distress and other measures

**Fig. 3:**
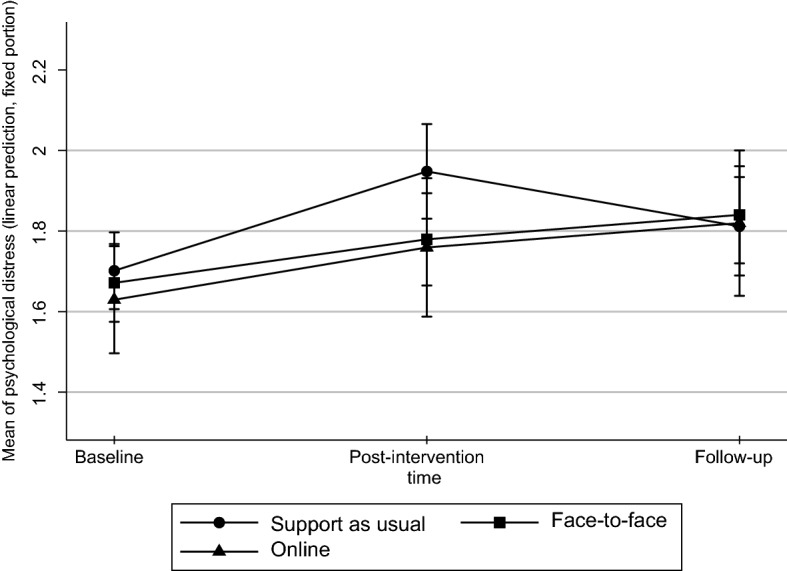
Intervention’s effects on psychological distress (CORE-OM) measured at baseline, post-intervention, and follow-up (Scale 1 = little distress. 5 = a lot of distress). Figures describe adjusted prediction of means with 95% confidence intervals

**Fig. 4:**
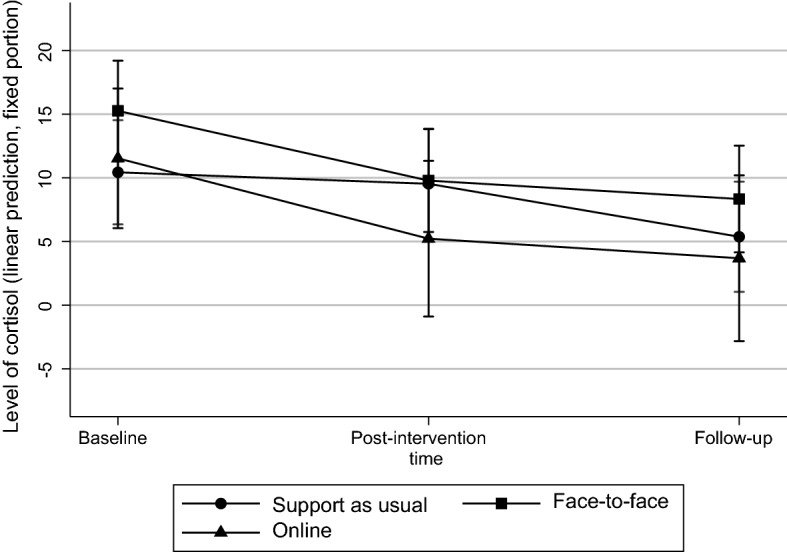
Intervention’s effects on level of cortisol measured at baseline, post-intervention, and follow-up (min. 0, max. 103,47). Figures describe adjusted prediction of means with 95% confidence intervals

Differences between the control and intervention groups in the primary and almost all the secondary outcomes were no longer apparent at follow-up (Tables 6 and 7, Figs. 3 and 4).

**Fig. 5:**
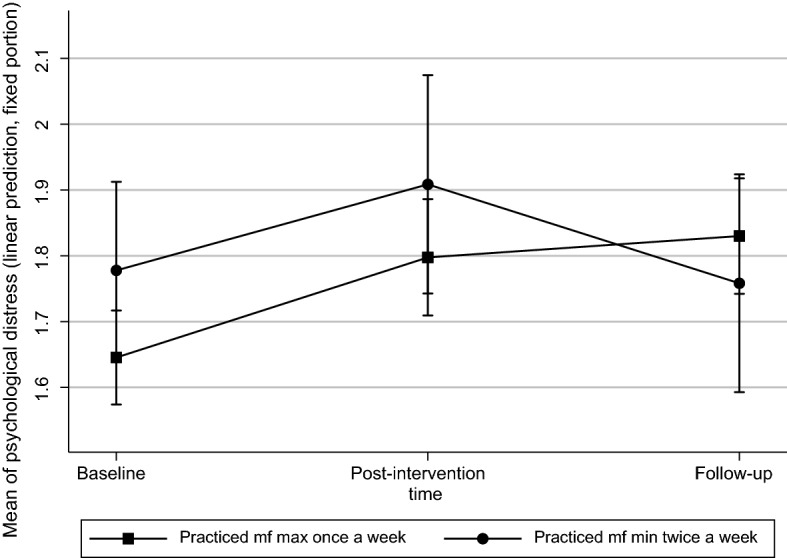
Effect of mindfulness practice on perceived distress (CORE-OM) at follow-up (Scale 1 = little distress. 5 = a lot of distress). Figures describe adjusted prediction of means with 95% confidence intervals

Further analyses were conducted to gain a deeper understanding of the longitudinal effects of the interventions and the study as a whole. All the participants (in both the control and the intervention groups) were divided into two groups based on how often they reported actively practising mindfulness during follow-up. Group 1 practised mindfulness at least twice a week, and Group 2 once a week at the most. The change in the level of perceived stress was different in these two groups. The participants who continued practising mindfulness at least twice a week were less stressed than the others. The difference between the two groups at baseline and follow-up was statistically significant (p = 0.035). (See Fig. 5).

### Immediate and longitudinal effects on mindfulness skills

The only secondary outcome measure on which the interventions had longitudinal effects was mindfulness skills. Mindfulness skills increased in the face-to-face mindfulness group during the intervention and remained at the higher level at follow-up. In the online ACT group, mindfulness skills remained almost the same at the three measurement points. In the control group, mindfulness skills decreased during the intervention but increased slightly at follow-up. Immediately after the intervention, the difference between the control group and the face-to-face group was significant (p < 0.001), but not between the control group and the online ACT group. At follow-up, similarly, the difference between the control group and the face-to-face mindfulness group (p = 0.018) was significant, but the difference between the control group and the online ACT group was not. (Supplement Fig. S1, and Supplement Tables S2 and S3).

The intervention had no reported adverse effects.

## Discussion

The aim of this study was to test whether two types of mindfulness intervention, face-to-face mindfulness training, and online ACT training could enhance students’ well-being and reduce their levels of stress. We examined the short- and long-term effects of mindfulness interventions on student stress and well-being and compared the effects of face-to-face and online mindfulness interventions on a control group. In addition, we explored how interventions motivated participants to practice mindfulness independently.

The Mindfulness Skills for Students course appeared to have helped the students maintain their functional ability and social interaction, reduce perceived study load, and prevent an increase in neuroticism immediately after the intervention and under increasing study pressure. The web-based Acceptance and Commitment Therapy intervention did not have such clear effects, but when the two intervention groups were pooled together the effect was about the same size as with the face-to-face mindfulness group alone.

Neither mindfulness interventions had immediate effect on the students’ quality of life, psychological flexibility, or resilience, or on their hair cortisol levels. The trend found in this study that cortisol levels decreased during the academic year regardless of intervention, requires further investigation.

The impact of the interventions on students’ stress and well-being seemed to disappear during the follow-up period. However, those students who continued practising mindfulness regularly, at least twice a week, were less stressed than other participants in follow-up. Both interventions motivated participants to practice mindfulness on their own during the course. Participants of the face-to-face mindfulness course practiced it more frequently and for longer at a time than participants of the online ACT course. In addition, an interesting finding was that almost one third of the control group practiced mindfulness during the intervention.

Our results are consistent with previous findings that mindfulness-based training prevent experiences of stress among health care students (Burton et al., 2017; McConville et al., 2017; Yamada & Victor, 2012). Our finding that neuroticism did not increase during the intervention as much in the intervention group as in the control group is in line with previous studies suggesting that mindfulness meditation may shape its practitioners toward healthier lifestyles (Armstrong & Rimes, 2016; Crescentini & Capurso, 2015; Rau & Williams, 2016). The practice of accepting and non-reactive self-reflection might ease negative and ruminative attitudes towards oneself, which is typical for highly neurotic personalities (Baer et al., 2006; Crane et al., 2016). Bellosta-Batalla and colleagues (2020) recently found that a brief mindfulness session reduced psychology students’ emotional turmoil and anxiety, and furthermore, elevated their salivary oxytocin levels. They concluded that mindfulness practice could foster an emotional and biological state of empathy, that could be addressed to either oneself or other people. More research with more participants is needed to find out the effects of ACT-based mindfulness intervention on health care students stress experience.

Evidence of longitudinal effects of mindfulness-based and ACT interventions is rare (de Vibe et al., 2013, 2018). In our study, comparison of the intervention and control groups showed that the effect of the interventions on students’ stress and well-being seemed to disappear during the follow-up period. However, when we examined in our follow up how regularly students practised mindfulness, we found that the students who continued practising it regularly, at least twice a week, were less stressed than the other participants. Based on our results, we argue that in order to reduce permanent stress, students should exercise mindfulness on a regular basis at least twice a week. Since our research showed that long-term practice of mindfulness is desirable, it would be interesting to investigate whether it can be supported, for example, by online reminders to students.

Our key finding was that when embedding mindfulness courses into the curriculum, it is important to support students’ motivation for regular individual practice. To initiate mindfulness practice, critical academic health care students need to obtain sufficient convincing research evidence of its positive effects. Motivation can be supported using evidence-based methods of health psychology, such as group support and the power of intrinsic motivation (Klein et al., 2015; Michie et al., 2005). The academic culture of students plays a crucial role in the acceptance of mindfulness. Mindfulness should be encouraged more widely in education and not only in certain mindfulness courses (Sottile, 2021), which would make it easier to keep up the practice.

According to a review by Martineau and his colleagues (2017), the reduction of students’ mental health problems requires a systematic and versatile approach. Mindfulness is an effective method, but alone it is not enough. Mindfulness training could be integrated into the subject courses, it could be offered as a separate or elective course, as part of the curriculum. Each institution needs to find their best way to incorporate it into its context. Mindfulness is neither the primary nor even a suitable stress management method for all health care students. The ‘spirit’ of mindfulness means that learning it should not be mandatory, and whereas participants should be invited to cultivate this ability.

Our research supports the view that mindfulness can be learned and instructed effectively in many ways. Face-to-face mindfulness instruction with weekly practice and discussions with the teacher and peers seemed to be more effective than mostly self-guided online learning. However, it is difficult to know whether this difference was due to the different programme and framework (Mindfulness-Based Stress Reduction vs Acceptance and Commitment Therapy), different teachers, or different group sizes (2 × 20 people vs 22 people). Overall, both interventions had a parallel impact on the students.

### Strengths and limitations

The strength of this study was that it was a high-quality intervention that rigorously implemented the principles of clinical trials. It had more than 50 participants and filled the quality gap detected by Galante et al. (2018); Pakenham and Stafford-Brown (2013), McConville et al. (2017), Burton et al. (2017), O’Driscoll et al. (2017). It adds to the knowledge on mindfulness training for students of dentistry, psychology, and logopaedics. All the questionnaires used were validated for the Finnish population, some especially for Finnish higher education students. The reliability of the primary outcome measure, its subscales, and most of the secondary outcome measures was good, except the personality trait of conscientiousness.

This study also had limitations. Three factors may have influenced our results, with the possible consequence that the difference between the intervention and the control group disappeared during follow-up. First, the participation in the trial was voluntary, and participants were particularly interested in mindfulness and stress reduction. Some of them informally reported that they were very keen to find out whether mindfulness helped them reduce stress. Most of the participants were enthusiastic at the beginning of the intervention and insisted on getting into the intervention groups. Many of those randomly selected for the control group were disappointed that they were unable to participate in the intervention. Even so, throughout the trial, the participants became strongly committed to the research project, as evidenced by the exceptionally high participation rate.

Second, we cannot rule out the possibility that contamination affected our results. The students were on the same campus and may have shared their experiences with each other. During the trial, some control group participants reported having started mindfulness or mindfulness-type practices on their own. In addition, some of them were friends and discussed the intervention during the trial. The trial might have been more valid if the control group had been in a different institution or at least on a separate campus, but then the heterogeneous stress exposure would have made comparison more difficult.

Third, since the online ACT group was significantly smaller than face-to-face mindfulness group, and it did not meet the pre-calculated power analyses, the results of online ACT intervention should be taken with more reservations than the results of the face-to-face mindfulness intervention.

Generalising the results to the entire academic student population in Finland and other countries should be done with caution. In our view, however, the results could be generalised to academic health care students in Finland, especially to psychology students who are not very stressed, are interested in stress management and aim to work with patients or clients.

In the future, it would be important to determine whether it is the regular practice of mindfulness per se that has an effect on stress reduction, or whether the effect requires some kind of conceptual change. Furthermore, the role of conceptual change in motivation to regularly practise should be further investigated. More detailed information is needed on the mechanisms through which mindfulness-based interventions affect health care students. Some recent, studies have shed light on this aspect by analysing qualitative data (Malpass et al. 2019; Weingarten et al. 2019). We also agree with Tang and Braver (2020) that more research is needed on how personality traits and individual differences affect responses to mindfulness interventions.

## Conclusion

This study was a randomised controlled trial that examined the short- and long-term effects of two different mindfulness interventions and a control group on students’ stress and well-being. The intervention groups were face-to-face Mindfulness Skills for Students program and online Student Compass program based on Acceptance and Commitment Therapy. Participants were students in medicine, dentistry, psychology, and logopaedics. We collected data at three measurement points (baseline, follow-up, and 4 months after follow-up) and used CORE-OM, which is a validated tool for examining the effects of psychosocial interventions and hair cortisol concentration as biological stress indicator. Our main finding was that the immediate differences between the primary outcomes of the control and intervention groups disappeared during follow-up. Face-to-face mindfulness intervention seemed to be more beneficial than the online Acceptance and Commitment Therapy based program. However, the students who continued to practise mindfulness regularly, at least twice a week, were less stressed than the other follow-up participants. Based on this result, we suggest that in order to reduce permanent stress through mindfulness, students should be motivated to practise it regularly.

## Supplementary Information

Below is the link to the electronic supplementary material.

Supplementary file1 (DOCX 30 kb)
