# Supplementary material for: Comparison of two different mindfulness interventions among health care students in Finland: a randomised controlled trial
Source: Adv Health Sci Educ Theory Pract. 2022 May 3;27(3):709–34. doi: 10.1007/s10459-022-10116-8 (PMC9063251; doi:10.1007/s10459-022-10116-8)
Supplement: Supplementary file 1 — Supplementary file1 (DOCX 30 kb) [file 10459_2022_10116_MOESM1_ESM.docx]

**Appendix**

### Supplement Figure 1. Intervention’s effect on mindfulness (FMI-13) at baseline, post-

### intervention and follow-up (Scale 1=little mindfulness. 5=a lot of mindfulness).

Figures describe adjusted prediction of means with 95% confidence intervals.

### Supplement Table 1: Comparison of stress and functional ability of intervention study participants and medical faculty students. Well-being study (conducted spring 2018).

|  | Participants of Intervention study  n=102 | | | Participants of Well-being study  n=845 | | |
| --- | --- | --- | --- | --- | --- | --- |
|  | M (SD) | Proportion of highly stressed (4–5) | Proportion of well-function (8–10) | M (SD) | Proportion of highly stressed (4–5) | Proportion of well-function (8–10) |
| Perceived stress* | 3.06 (0.91) | 29.4% |  | 3.45 (1.06) | 49.7% |  |
| Functional ability in studies ** | 7.77 (1.25) |  | 63.7% | 7.06 (2.34) |  | 54.6% |

* Scale: 1=not at all, 5=very much ** Scale 0–10

### Supplement Table 2. Number of items, scales, Chronbach’s Alphas, means, standard deviations and references of secondary outcomes of three intervention groups at baseline, post-intervention and follow-up.

|  | Questionnaire | | | Baseline | Post-intervention | Follow-up |
| --- | --- | --- | --- | --- | --- | --- |
|  | No of items | Scale | Alpha | Mean (SD) | Mean (SD) | Mean (SD) |
| Freiburg Mindfulness Inventory | 13 | 1-4 | 0.8281 |  |  |  |
| Support as usual |  |  |  | 2.58 (0.51) | 2.39 (0.48) | 2.59 (0.60) |
| Face-to-face |  |  |  | 2.44 (0.43) | 2.64 (0.40) | 2.62 (0.46) |
| Online |  |  |  | 2.65 (0.32) | 2.65 (0.41) | 2.59 (0.42) |
| Psychological flexibility  for higher education students (Asikainen et al., 2017) | 7 | 1–5 | 0.8852 |  |  |  |
| Support as usual |  |  |  | 3.44 (0.73) | 3.40 (0.66) | 3.42 (0.65) |
| Face-to-face |  |  |  | 3.51 (0.57) | 3.60 (0.64) | 3.66 (0.59) |
| Online |  |  |  | 3.31 (0.62) | 3.42 (0.78) | 3.43 (0.57) |
|  |  |  |  |  |  |  |
| Study load adopted from the healthy work questionnaire (Karasek & Theorell, 1990) | 9 | 1–5 | 0.7252 |  |  |  |
| Support as usual |  |  |  | 1.92 (0.51) | 2.23 (0.51) | 2.04 (0.55) |
| Face-to-face |  |  |  | 1.93 (0.62) | 1.99 (0.59) | 2.12 (0.63) |
| Online |  |  |  | 1.95 (0.49) | 2.27 (0.67) | 2.13 (0.43) |
|  |  |  |  |  |  |  |
| Perceived stress (Lundqvist ja Mäkiopas, 2016) | 1 | 1–5 | - |  |  |  |
| Support as usual |  |  |  | 3.08 (0.90) | 3.63 (1.00) | 3.45 (0.87) |
| Face-to-face |  |  |  | 3.13 (0.88) | 3.13 (0.88) | 3.44 (0.86) |
| Online |  |  |  | 2.9 (0.97) | 3.5 (0.97) | 3.2 (0.68) |
|  |  |  |  |  |  |  |
| Perceived recovery (Elo et al. 1992) | 1 | 1–5 | - |  |  |  |
| Support as usual |  |  |  | 2.41 (0.94) | 2.66 (0.97) | 2.48 (0.97) |
| Face-to-face |  |  |  | 2.55 (0.80) | 2.5 (0.83) | 2.74 (1.02) |
| Online |  |  |  | 2.5 (1) | 2.63 (0.89) | 2.9 (0.88) |
|  |  |  |  |  |  |  |
| Functional ability in studies modified from the measure of functional ability at work (Tuomi et al., 1998) | 1 | 0–10 | - |  |  |  |
| Support as usual |  |  |  | 7.72 (1.40) | 7.31 (1.41) | 7.58 (1.64) |
| Face-to-face |  |  |  | 7.76 (1.05) | 8 (1.16) | 7.56 (1.81) |
| Online |  |  |  | 7.75 (1.48) | 7.38 (1.86) | 8.2 (1.26) |
|  |  |  |  |  |  |  |
| Perceived quality of life (Koivumaaa ym, 2000; Allardt 1973; Lundqvist ja Mäkiopas, 2016) | 1 | 1–5 | - |  |  |  |
| Support as usual |  |  |  | 4.02 (0.67) | 3.94 (0.54) | 4.06 (0.75) |
| Face-to-face |  |  |  | 4.08 (0.49) | 4.08 (0.49) | 4.09 (0.63) |
| Online |  |  |  | 4 (0.56) | 4.19 (0.40) | 4 (0.53) |
|  |  |  |  |  |  |  |
| Warwick–Edinburgh Mental Well-being Scale (Putz et al., 2012; Stewart-Brown & Janmohamed, 2008; Stewart-Brown et al., 2009) | 7 | 1–5 | 0.7426 |  |  |  |
| Support as usual |  |  |  | 3.74 (0.43) | 3.49 (0.45) | 3.64 (0.45) |
| Face-to-face |  |  |  | 3.65 (0.38) | 3.59 (0.42) | 3.59 (0.50) |
| Online |  |  |  | 3.78 (0.42) | 3.75 (0.55) | 3.75 (0.40) |
|  |  |  |  |  |  |  |
| Personality trait: neuroticism, part of Big five (Lang et al., 2011); | 3 | 1–5 | 0.8296 |  |  |  |
| Support as usual |  |  |  | 3.33 (0.87) | 3.61 (0.79) | 3.36 (0.96) |
| Face-to-face |  |  |  | 3.24 (0.92) | 3.11 (0.91) | 3.04 (0.97) |
| Online |  |  |  | 3.33 (0.80) | 3.58 (1.01) | 3.57 (1.04) |
|  |  |  |  |  |  |  |
| Personality trait: conscientiousness, part of Big five (Lang et al., 2011); | 3 | 1–5 | 0.5326 |  |  |  |
| Support as usual |  |  |  | 4.03 (0.51) | 3.96 (0.52) | 4.09 (0.53) |
| Face-to-face |  |  |  | 4.05 (0.52) | 3.99 (0.64) | 4.02 (0.67) |
| Online |  |  |  | 4.1 (0.52) | 3.79 (0.63) | 4.24 (0.61) |
|  |  |  |  |  |  |  |
| Resilience modified version of the Resilience scale (Wagnild & Young, 1993; Losoi et al., 2013) | 5 | 1–7 | 0.7369 |  |  |  |
| Support as usual |  |  |  | 5.63 (0.73) | 5.46 (0.96) | 5.62 (0.89) |
| Face-to-face |  |  |  | 5.72 (0.71) | 5.72 (0.77) | 5.65 (0.79) |
| Online |  |  |  | 5.72 (0.70) | 5.56 (0.74) | 5.27 (1.14) |
| Evaluation of ones’ own health (Kunttu et al., 2017) | 1 | 1–5 (1=good, 5=bad) | - |  |  |  |
| Support as usual |  |  |  | 1.62 (0.67) | 1.86 (0.69) | 1.76 (0.83) |
| Face-to-face |  |  |  | 1.61 (0.68) | 1.53 (0.65) | 1.65 (0.69) |
| Online |  |  |  | 1.55 (0.60) | 1.75 (0.68) | 1.87 (0.74) |

### Supplement Table 3. P-values of repeated measures mixed model, immediate and longitudinal effects secondary outcomes

|  | Immediate effect | Longitudinal efect |
| --- | --- | --- |
| **Freiburg Mindfulness Inventory** |  |  |
| Support as usual vs. face-to-face | 0.000 | 0.018 |
| Support as usual vs. online | 0.085 | 0.839 |
| Support as usual vs. both interventions | 0.000 | 0.067 |
| **Psychological flexibility for higher education students** |  |  |
| Support as usual vs. face-to-face | 0.307 | 0.187 |
| Support as usual vs. online | 0.484 | 0.395 |
| Support as usual vs. both interventions | 0.271 | 0.165 |
| **Study load** |  |  |
| Support as usual vs. face-to-face | 0.056 | 0.712 |
| Support as usual vs. online | 0.837 | 0.702 |
| Support as usual vs. both interventions | 0.122 | 0.904 |
| **Perceived stress** |  |  |
| Support as usual vs. face-to-face | 0.020 | 0.781 |
| Support as usual vs. online | 0.856 | 0.765 |
| Support as usual vs. both interventions | 0.102 | 0.755 |
| **Perceived recovery** |  |  |
| Support as usual vs. face-to-face | 0.124 | 0.581 |
| Support as usual vs. online | 0.505 | 0.395 |
| Support as usual vs. both interventions | 0.148 | 0.435 |
| **Functional ability in studies** |  |  |
| Support as usual vs. face-to-face | 0.037 | 0.973 |
| Support as usual vs. online | 0.951 | 0.159 |
| Support as usual vs. both interventions | 0.110 | 0.530 |
| **Perceived quality of life** |  |  |
| Support as usual vs. face-to-face | 0.512 | 0.958 |
| Support as usual vs. online | 0.091 | 0.860 |
| Support as usual vs. both interventions | 0.231 | 0.969 |
| **Warwick–Edinburgh Mental Well-being Scale** |  |  |
| Support as usual vs. face-to-face | 0.076 | 0.911 |
| Support as usual vs. online | 0.141 | 0.713 |
| Support as usual vs. both interventions | 0.053 | 0.833 |
| **Personality trait: neuroticism, part of Big five** |  |  |
| Support as usual vs. face-to-face | 0.013 | 0.399 |
| Support as usual vs. online | 0.937 | 0.446 |
| Support as usual vs. both interventions | 0.068 | 0.744 |
| **Personality trait: conscientiousness, part of Big five** |  |  |
| Support as usual vs. face-to-face | 0.732 | 0.478 |
| Support as usual vs. online | 0.140 | 0.367 |
| Support as usual vs. both interventions | 0.271 | 0.165 |
| **Resilience** |  |  |
| Support as usual vs. face-to-face | 0.223 | 0.852 |
| Support as usual vs. online | 0.527 | 0.166 |
| Support as usual vs. both interventions | 0.233 | 0.471 |
| **Evaluation of ones’ own health** |  |  |
| Support as usual vs. face-to-face | 0.057 | 0.612 |
| Support as usual vs. online | 0.707 | 0.455 |
| Support as usual vs. both interventions | 0.169 | 0.734 |
